# Amputation of the Lower Extremities for Gangrene from Frost-bite

**Published:** 1882-05-20

**Authors:** Thomas F. Houston

**Affiliations:** Clarksville, Ga.


### Southern Medical Record:

EDITORS:

T. S. POWELL, M.D. W. T. GOLDSMITH, M.D. R. C. WORD, M.D.

*R. C. WORD, M.D., Managing Editor.*

☞All Communications and Letters on Business connected with the RECORD must be addressed to the Managing Editor.

VOL. XII. ATLANTA, GA., MAY 20, 1882. NO. 5.

### ORIGINAL AND SELECTED ARTICLES.

### AMPUTATION OF THE LOWER EXTREMITIES FOR GANGRENE FROM FROST-BITE.

BY THOMAS F. HOUSTON, M. D., CLARKSVILLE, GA.

On the 19th of November I was summoned to testify as a medical expert in the case of John Parker (colored), a prisoner awaiting trial for stealing a watch. On reaching the jail I found him lying on the floor sullenly resisting all attempts to dress him. From the sheriff I learned that at 8 a. m., only two hours before, he was perfectly sane; asked the prospect of reaching his case that day, and expressed his desire for a speedy trial. That he ate his breakfast with evident relish, and laughed and talked with the other prisoners. When he went down to the jail to bring him to the court-house, the prisoner was standing sullenly in the corner perfectly naked, and not only refusing to speak, but showed decided tendency to fight all who came in his reach, whereupon I was immediately summoned. I examined him and could find absolutely nothing abnormal about him. So I directed him to be dressed in spite of his violent struggles, and carried *vi et armis* to the court-house. On the stand I gave as my opinion (based upon eleven years' residence at the Georgia State Asylum) that he was a malingerer. What confirmed my suspicion was the fact that when he was conscious of being watched he would attempt to show decided evidences of insanity—there was a want of uniformity in his manner of "playing crazy." One moment he would lie like one dead, breathing normally; the next he would strike at all in reach of him and roar like a madman. When he believed himself unobserved he would set up and act like a perfectly rational being.

On my opinion he was tried, convicted and sentenced to hard labor for one year. During the trial he lay like a dead man upon the floor, and in such a condition was carried by four men to the jail. As they laid him down he jumped up, struck his heels together andr emarked: "Well, I am guilty, but I would have fooled the law but for that damned Doctor." In one hour he had torn off his clothes and thrown them out of the window, and was acting in the same manner again.

On December 1st I was requested to see him and decide if he could be removed to the penitentiary. I again examined him, having learned that since the day after the trial, he had not spoken nor gotten out of his bed, which emitted an intolerable odor from the excretions he had voided in it.

*Examination.*—Pupils normal, pulse 108, full and hard; percussion and auscultation found no abnormality. No perceptible tenderness in any portion of the body; muscles responded normally to electricity, tongue clean. Found four large blisters, two on each ankle, about 2 inches in diameter, occupying the site of some old shackle marks that he had worn during some former service in the penitentiary. The blisters were evidently the effects of two cold nights which had occurred during the time that he was attempting to play the madman—when the thermometer fell to about 13° F. The blisters were filled with dark-yellow serum. Such was the rascal's fortitude that the strongest current of a No. 4 Galvano-Faradic battery would not elicit a wince other than the natural muscular contraction. As the convict had always borne a reputation for intelligence and desperate recklessness, I again decided that it was "pure cussedness," as they say in the Southwest. But the prison authorities could not induce him, by threats or persuasion, to show the slightest sign of human intelligence, so they had to let him lie in his filth.

On the following day the Ordinary had the prisoner removed to the dwelling of another negro, and left him in his charge. Some days after the nurse came for a bottle of "Darby's Prophylactic Fluid" to use in bathing his ankles, stating that they were getting in a bad condition.

On December 8th I was summoned to see him. On entering the room I remarked to a medical student who accompanied me: "I smell gangrene." Upon uncovering his feet a most horrible sight greeted my eyes. He was suffering from gangrene of both extremities; the skin had slipped off like a sock. The toes were wanting, the lower row of meto-tarsal bones protruded through the flesh fully ½ inch, and in several other places the flesh had fallen off, revealing the tarsal bones; the diseased action extended back as far as the maleoli. Temperature, 103° F. Respiration 24. Pulse, 120, feeling like a cord under the finger. Prescribed elix. beef, wine and iron, egg-nog and Valentine's meat-juice, and the Saline antimonial mixture:

| | | |
|---|---|---|
| ℞ | Sulph. magnesia | ℥i, |
| | Tr. verat. viride | ʒi, |
| | Sulph. quinine | grs. xx, |
| | Aromat. sulph. acid | ʒii ss, |
| | Laudanum | ʒii, |
| | Aqua dis. q. s. ft | ℥viij. M. |

S. Tablespoonful the first hour, one teaspoonful each subsequent hour until temperature falls to 99° F., the dose to be increased pr. r. n.

The feet to be bathed every hour with a solution of chloride of lime. Gave calomel, grs. v.

December 10th. Temperature 101 F.; pulse, 110; action dark and loose; gave the same treatment, except the calomel.

December 11th. Temp. 100 F.; urine bloody; pulse, 99, small and steady. Gave liquor potas and spirits of nitre equal parts, *ter die;* increased the whisky.

December 12th. Temp. 100 F.; pulse, 98, stronger; urine normal in color and not so acid. Actions still very dark; repeated the calomel.

It is not necessary to continue the daily record. It is sufficient to say the saline anti-mix. kept the temperature about 100° F., and the pulse about 90. I became satisfied that the patient's strength would not last until the line of demarkation was fully established, and when it became evident that the diseased action would not probably extend above the middle third, I determined to operate without waiting. And so, on the 9th day of the treatment I amputated both of his legs in the upper third. Being assisted by Drs. Phillips and Rossignole. When Dr. P. attempted to give him chloroform he resisted as best he could, swearing that he had rather die than lose his legs. Not considering, in his mental and physical condition, that he was able to decide this question, and as the county authorities urged the operation, I paid no attention to his remonstrances. I used Esmarch's bandage and Petit's tourniquet in addition to the elastic cord. I chose the circular method, and the cabin being badly lighted moved the patient out into the sunshine.

The thermometer stood at 40° F., and cool air, aided by quantities of ice-water, was a great factor in preventing oozing after the arteries were tied. I did not find it necessary to ligate the veins. The loss of blood did not amount to more than four ounces. He reacted splendidly, and the external edges of the flaps united by first intention. I had to keep the lower angle open to insure good drainage. Had the stumps injected frequently with the weak solution of chloride of lime. Controled the pulse and temperature by appropriate means, and gave the same supporting treatment I had previously employed. He did splendidly for two weeks, when he had a very sharp attack of pleurisy, complicated with bronchitis. At length this trouble also was removed, and to-day I sent him home, having obtained a pardon from the Governor.

The prisoner is now grateful to me for the efforts I made in his behalf, and acknowledges that he was shamming.

Before closing, I will relate a somewhat similar case of malingering that occurred at the Georgia State Lunatic Asylum during the administration of the lamented Dr. Thomas F. Green. He was summoned to see ————, a white convict, sentenced to ten years for stealing.

The day after he was admitted to the penitentiary he became suddenly a perfect imbecile. Dr. Green found him sitting on the ground, his lower jaw relaxed, the saliva dripping upon his breast, his eyes set in a vacant stare; and his only reply to any question was, "Let me go home with you," drawled out through his nose. Offer him a plug of tobacco and he would snap at it like a dog, and swallowing it in great mouthfuls, evincing not the slightest nausea. During the thirty-four years that Dr. Green was in charge of the asylum, he was often called upon to testify as a medical expert, and in not a single instance did subsequent developments reverse his decision. After a careful examination he decided that the man was shamming, and so informed the prison authorities.

Dr. Green determined to force him to acknowledge his fraud; and having summoned a trusty attendant directed him to prepare a room, placing a wire screen across the window, so that plenty of air and a little light would be admitted, but the inmate could not see out into the yard. In the presence of the impostor he gave the following instructions: "This man is pretending to be insane. He is sentenced to the penitentiary for ten years. He has been sent to me, and I intend to keep him in this room for the next ten years.

Do not speak to him, or allow any one else to do so; never visit him except to carry his meals and water; and under no circumstances allow any one to see him or permit him to come out of his room. I have condemned him to solitary confinement for ten years."

The malingerer stood it for three days; on the fourth morning he besought the attendant to summon Dr. Green, exclaiming, "D—n it, I want to go back to the penitentiary; tell Dr. Green I want to see him." He said upon seeing the Doctor, "Oh, I am well again. I felt my insanity, like a cold wave, go down my back and out at my heels. Your treatment cured me." With a dry smile Dr. Green answered, "I thought it would." He had him returned to the prison authorities, and a more obedient and faithful convict was never in the Georgia penitentiary.

